# Elevated levels of mental health issues among optometrists in the United Kingdom

**DOI:** 10.1111/opo.13553

**Published:** 2025-07-17

**Authors:** Neil Retallic, Lindsay Rountree, Sharon Bentley, Fiona Fylan, David B. Elliott

**Affiliations:** ^1^ School of Optometry and Vision Science University of Bradford Bradford UK; ^2^ Herbert Wertheim School of Optometry and Vision Science University of California Berkeley Berkeley California USA; ^3^ Leeds Sustainability Institute Leeds Beckett University Leeds UK

**Keywords:** depression, distress, mental health, optometry, practitioner, well‐being

## Abstract

**Purpose:**

To assess the mental health and well‐being of optometrists in the UK and explore associated factors.

**Method:**

A cross‐sectional online survey of UK‐based optometrists was conducted over a 3‐month period during 2024. The survey included validated, well‐established measures to assess psychological distress (Kessler Psychological Distress Scale), depression (Patient Health Questionnaire‐2) and anxiety (General Anxiety Disorder‐2). Well‐being was evaluated in relation to optimal healthy living behaviours.

**Results:**

The study includes results from 1303 qualified optometrists, of whom 37% had moderate‐to‐severe psychological distress scores, 24% screened positive for depression and 28% for anxiety. The three strongest predictors of higher psychological distress in a regression analysis were younger age, lower self‐reported physical health and the absence of additional roles. For both depression and anxiety, the significant predictors were younger age, poorer self‐reported physical health and not being an Independent Prescriber. Across all three mental health measures, female optometrists exhibited poorer scores than male optometrists. Adherence to healthy lifestyle behaviours was associated with better mental health outcomes. However, neither gender nor adherence to healthy lifestyle behaviours was identified as independent predictors and was therefore not retained in the final logistic regression model.

**Conclusion:**

Higher prevalence of mental health conditions was observed among UK‐based optometrists than in the general population. Optometrists with higher qualifications and those undertaking additional roles may experience improved mental well‐being, although further research is needed. The findings emphasise the need for initiatives to support mental well‐being, particularly among young optometrists and those early in their optometry career.

**Key points**

- This study identified elevated levels of psychological distress, depression and anxiety among optometrists in the United Kingdom, with not all individuals seeking professional support or treatment.
- Risk factors for poorer mental well‐being included being young and having lower self‐rated physical health. Better mental health was associated with having higher qualifications, additional roles and healthier lifestyle choices.
- The profession should implement targeted mental well‐being initiatives, with a particular focus on those most at risk.

## INTRODUCTION

Mental health disorders impact nearly a billion people worldwide^1^ and are recognised as a leading cause of disability.^2^, ^3^ In the UK, approximately one in five people report symptoms of a common mental health condition, such as depression or anxiety.^4^ Mental health encompasses a broad range of psychological states that contribute to an individual's ability to cope with the normal stresses of life, work productively and engage meaningfully with their community.^1^ Psychological distress, depression and anxiety are mental health conditions that are particularly prevalent among healthcare professionals,^5^, ^6^, ^7^, ^8^, ^9^, ^10^, ^11^, ^12^, ^13^ including optometrists.^14^, ^15^ Occupational stressors for optometrists include complex clinical decision‐making,^16^, ^17^, ^18^ working with patients^14^ and expanding scopes of practice.^17^ Retail‐related pressures exist within optometry^14^ that are not commonly encountered in other healthcare professions. The manslaughter conviction of a UK optometrist (Honey Rose) for missing papilloedema in a child^19^ led to uncertainty and increased defensive clinical decision‐making within the UK optometry profession,^19^ contributing to a systemic concern about litigation.^20^

However, there is limited research on the mental health status of practising optometrists and none from within the UK. A pre‐COVID‐19 2021 study of 505 Australian optometrists found that nearly one in five (21%) had received treatment for a mental health condition in the previous year, with nearly one in three reporting moderate‐to‐severe psychological distress (31%), depression (30%) and anxiety (31%).^14^ Similarly, a 2024 study of 2124 ophthalmic personnel that included 359 optometrists in the USA and Canada reported a high prevalence (38%) of probable depression, anxiety or both during the COVID‐19 pandemic.^15^ A 2025 study of Fellows of the American Academy of Optometry reported that 51% of 321 fellows reported high levels of stress, 9% screened positive for anxiety and 4% for depression. Notably, 46% of these were academics.^21^

Physical health and lifestyle factors are intrinsically linked to mental health, influencing overall well‐being and psychological health.^22^ Self‐reported physical health has been associated with mental health status^23^ and well‐being measures.^22^ Therefore, understanding the mental health status of optometrists also requires consideration of lifestyle factors that contribute to overall health and well‐being. Recommended guidelines include consuming a healthy diet of at least five portions (or 400 g) of fruit and vegetables per day,^24^ undertaking at least 150 min of moderate aerobic activity or 75 min of vigorous activity per week, or a combination of both,^25^ not smoking and limiting alcohol consumption.^26^

Given the high prevalence of mental health conditions among healthcare professionals^5^, ^6^, ^7^, ^8^, ^9^, ^10^, ^11^, ^12^, ^13^ and the challenges within optometry, this study aims to address a critical gap by assessing the mental health of UK optometrists using validated measures of psychological distress, depression and anxiety. Additionally, we sought to determine whether the risk factors for poorer mental health outcomes found in the general population, including gender, age and lifestyle behaviours, are associated with poorer mental health outcomes among UK optometrists.

## METHODS

A cross‐sectional survey of qualified optometrists registered with the General Optical Council^27^ was conducted from April to July 2024. Participants were recruited through advertisements at national optometry conferences, social media, email communications from professional optical organisations, two optometry magazines, educational events and word of mouth. Eligibility criteria required participants to be delivering UK patient‐facing eye care services for a minimum of 4 h per week. Consent details were covered in the introductory information, and ethical approval for the study (EC28194) was granted by the Chair of the Biomedical, Natural, Physical and Health Sciences Research Ethics Panel at the University of Bradford.

### Survey

Participants accessed the online Microsoft Form survey via a web link or QR code. The survey comprised questions on several aspects of mental health and well‐being.

The goal was to develop a brief survey (to encourage participation and completion), incorporating instruments with demonstrated validity in clinical and research settings with healthcare professionals.^14^, ^28^ In selecting the instruments, consideration was also given to availability of normative UK population data for comparison,^29^, ^30^ and alignment with national mental health screening guidelines.^31^, ^32^ We also considered a variety of questions about the workplace and optometrists' roles that could highlight risk factors for poor mental health among UK optometrists. Pilot data of the survey from 32 optometrists showed a mean completion time of 7.5 min.

### Demographics and workplace characteristics

We included demographic questions (age, gender and years since qualification), which have been shown to affect mental health in previous studies.^14^, ^29^, ^33^ The following workplace factors, considered to be potential risk factors for mental health problems, were also included: country of workplace within the UK (due to differing remuneration models for optometry in different countries),^34^ scope of practice (Independent Prescriber [IP], who are able to use therapeutic management of eye disease vs non‐IP), primary work setting (independent vs multiple vs hospital vs locum), additional roles (e.g. supervising trainees, people management, teaching and advisory or industry) and weekly working hours.

### Mental health scales

The Kessler Psychological Distress Scale (K10),^35^ Patient Health Questionnaire‐2 (PHQ‐2)^36^ and Generalized Anxiety Disorder‐2 (GAD‐2)^37^ were selected to assess psychological distress, depression and anxiety, respectively. Normative post‐COVID‐19 data were available for the adult UK population (*N* = 987, age range 18–86 years).^29^

The K10 scale provides a general measure of mental health and is widely used due to its ability to categorise individuals by clinically significant levels of non‐specific mental distress with good predictability.^35^, ^38^ The K10 is well established and has been included in numerous epidemiological studies by governments^38^, ^39^, ^40^, ^41^ and the World Health Organization (WHO),^35^ as well as a study of optometrists in Australia.^14^ The scale comprises 10 questions where respondents rate their feelings over the past 30 days on a 5‐point Likert scale (from ‘none of the time’ to ‘all of the time’). Scores are summed to give a total out of 50 and categorised as follows: low or no psychological distress (10–19), mild distress (20–24), moderate distress (25–29) and severe distress (30–50).^35^ The K10 is preferred over alternatives due to its strong association with diagnostic criteria, its specific design for general population screening, its validity and its broad applicability.^35^

The PHQ‐2 and GAD‐2 are brief, validated screening tools for depression and anxiety, respectively, widely used in UK primary care and research.^31^, ^37^, ^42^, ^43^, ^44^, ^45^ Both scales align with the Diagnostic and Statistical Manual of Mental Disorders (DSM‐5) criteria for major depressive disorder^46^ and generalised anxiety disorder.^46^ The PHQ‐2 consists of two questions assessing the frequency of low mood and anhedonia over the past 2 weeks, reflecting key indicators of depression.^36^ The GAD‐2 comprises two questions focusing on feeling nervous, anxious or on edge and difficulty controlling worry.^47^ These measures share the same response format, scoring system (0–6) and a threshold of ≥3 for identifying individuals at risk.^36^, ^43^, ^45^ They are also endorsed by the National Institute for Health and Care Excellence (NICE) as first‐step screening tools.^31^

### Factors influencing mental health

To assess current mental health status, participants were asked whether they had consulted a healthcare professional about a mental health condition within the past 12 months, and if so, whether they had received treatment. This enabled comparison with the Australian optometrists study.^14^ Respondents were also asked to self‐rate their physical health on a scale from 1 to 10, with 1 indicating very unhealthy and 10 indicating very healthy, an approach similar to that taken by other studies.^28^, ^48^

### Lifestyle behaviours

Health‐promoting lifestyle questions were included, with a yes/no response format based on the WHO healthy living guidelines^24^ and UK national guidance.^25^, ^49^ These questions asked whether respondents typically consume at least five portions of fruit and/or vegetables per day, engage in at least 150 min of moderate physical activity or at least 75 min of vigorous activity, use nicotine in any form, and/or consume alcohol four or more times per week.

### Participants

At least one of the three validated questionnaires was completed by 1303 optometrists who met the eligibility criteria for this study. This represented 7% of the UK General Optical Council (GOC) register of optometrists^27^ as of May 2024, a sample broadly representative of the demographic distribution (Table 1). The gender distribution (67% female, 33% male) was similar to the GOC register (62% female, 38% male). Mean age was 40 years (SD = 11.1, range: 22–77 years).

**TABLE 1:** Demographic characteristics of optometrist respondents compared with the General Optical Council (GOC) register of UK optometrists.

Characteristics	Number of respondents	Representation to UK GOC optometrist data base (%)
**UK optometrists**	**1303**	**7**
*Gender*		
UK female optometrists	870	8
UK male optometrists	420	6
*Country*		
Optometrists in England	844	6
Optometrists in Wales	190	24
Optometrists in Scotland	206	12
Optometrists in Northern Ireland	63	10
*IP status*		
UK non‐IP optometrists	1056	7
UK IP optometrists	238	13

*Note*: Some categories include missing data, resulting in variable total numbers. All respondents were UK‐based, with a total sample size of *N* = 1303.

Abbreviation: IP, independent prescriber.

The distribution across UK countries was similarly representative, with minor overrepresentation from Wales and Scotland. Gender distribution showed a consistent female‐to‐male ratio across regions. In terms of workplace types, around half (54%) worked primarily in multinational chain practice(s), 22% in independent practice(s), 13% as self‐employed locums, 9% in hospitals and 2% in other settings, such as academia. Most optometrists worked full‐time (63%), averaging 35.2 h per week (SD = 10.0 h).

All three questionnaires were completed by 1284 optometrists who participated (99%). If a participant did not respond to any questions in a questionnaire, these data were excluded from the analysis of that questionnaire.

### Analysis

Statistical analysis was conducted using IBM SPSS Statistics version 29.0 (ibm.com). All mental health measures (K10, PHQ‐2 and GAD‐2 scores) and physical health ratings were non‐normative, as confirmed by the Kolmogorov–Smirnov Goodness of Fit test. Non‐parametric tests were applied, including the Mann–Whitney U test for binary outcomes and the Kruskal–Wallis H test for multiple outcome options, to identify statistically significant differences between variables. A significance threshold of *p* < 0.05 was set. As such, the median and interquartile range are reported. As mean and standard deviation for these measurements are commonly reported in similar publications,^14^, ^15^, ^29^ these have also been included for comparative purposes. Free‐text numerical responses on working hours were dichotomised for analysis into part‐time (<35 h per week) and full‐time (≥35 h per week).^50^ Year of qualification was dichotomised into early career (≤5 years) versus more experienced (>5 years), consistent with previous research.^51^

Logistic regression with a backward stepwise approach was employed to determine risk factors for moderate‐to‐severe psychological distress (K10), positive screening for depression (PHQ‐2) and positive screening for anxiety (GAD‐2). The independent variables included in the analyses were age, gender, work location, scope of practice, working hours, additional roles, physical health rating and the four healthy lifestyle behaviours. Variables such as years since qualification (i.e. overlapping with age) and other mental health measure results were excluded to meet the assumptions of logistic regression. The most parsimonious model was determined by retaining variables with the strongest associations, using an initial inclusion criterion of *p* < 0.1, which was subsequently refined to *p* < 0.05.

For nominal data comparisons, which included the healthy lifestyle behaviours and consulting a healthcare professional for mental health issues, Pearson Chi‐squared tests were used to test for significance, with a threshold of *p* < 0.05.

Comparisons to the UK adult population (*N* = 987) were made using data from a normative population study,^29^ as well as with findings from other studies with optometrists.^14^, ^15^ Since only mean, standard deviation and sample size data were available in these studies, we conducted Welch t‐tests on mean scores from the mental health screening tools to compare their data with that from our study and identify statistically significant differences. Comparisons to external studies were incorporated in the discussion section rather than the analysis, in order to maintain a clear distinction between the primary data analysis and the broader contextual interpretation.

## RESULTS

### Mental health scales

**FIGURE 1:**
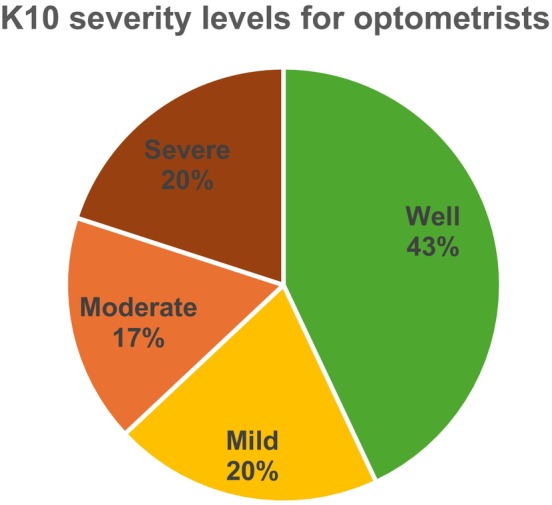
Severity levels of psychological distress among optometrists, measured using the Kessler Psychological Distress Scale (K10). *N* = 1291. Categories are defined as: Well (K10 score 10–19), mild (20–24), moderate (25–29) and severe (30–50).

Although 43% of participating optometrists were classified as ‘well’ (K10 scores ≤19, Figure 1), 37% were identified as experiencing moderate‐to‐severe psychological distress (K10 score ≥ 25), while 24% screened positive for depression (PHQ‐2 score ≥3) and 28% for anxiety (GAD‐2 score ≥3).

### Factors influencing mental health

One in five participating optometrists (21%) reported consulting a healthcare professional for mental health concerns in the past year. Of these, the majority (81%) received treatment, which represented 17% of the total cohort. There were no statistically significant differences between optometrists across countries, gender, workplace, pattern of work or IP status. Among optometrists who had not sought support from a healthcare professional, 31% had a moderate‐to‐severe K10 score, while 20% met the threshold for depression (PHQ‐2 ≥3) and 23% for anxiety (GAD‐2 ≥3), warranting further evaluation with a healthcare professional.

For self‐ratings of physical health, the mean score for participating optometrists was 7.1 (SD = 1.7), and the median (Q1–Q3) was 7 (6–8).

### Healthy lifestyle behaviours

**FIGURE 2:**
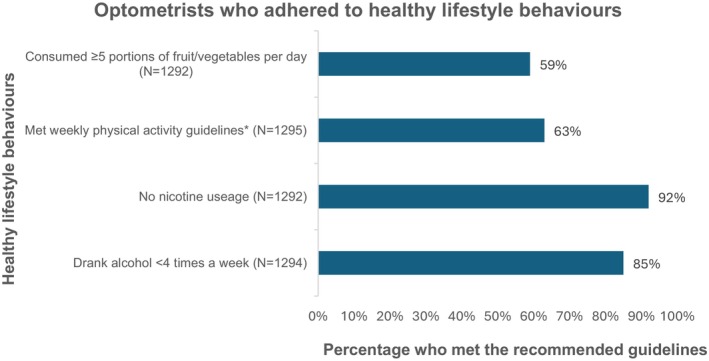
Healthy lifestyle behaviours among optometrists. The percentage values indicate the proportion of optometrists who met the recommended requirements for each healthy lifestyle behaviour out of the total respondent numbers (N values). *Guidelines are ≥150 min of moderate or ≥75 min of vigorous physical activity per week.

The percentages of optometrists who met the recommended healthy lifestyle behaviours are shown in Figure 2, with 33% reporting adherence across all four behaviours. Of the four behaviours, the lowest adhesion rate was observed for consuming at least five portions of fruit and vegetables daily (59%) while the majority of participants reported being a non‐smoker (92%).

Male optometrists were significantly more likely to exercise, *X* ^2^ (1, *N* = 1282) = 4.82, *p* = 0.03, to be smokers, *X* ^2^ (1, *N* = 1279) = 11.78, *p* < 0.001 and to consume more alcohol, *X* ^2^ (1, *N* = 1281) = 25.30, *p* < 0.001 than female optometrists. IPs demonstrated healthier behaviours than non‐IP optometrists, particularly in fruit and vegetable consumption, *X* ^2^ (1, *N* = 1283) = 9.47, *p* = 0.002, exercising more, *X* ^2^ (1, *N* = 1286) = 7.97, *p* = 0.005 and smoking less, *X* ^2^ (1, N = 1283) = 6.27, *p* = 0.01. However, IP optometrists were more frequent drinkers of alcohol, *X* ^2^ (1, *N* = 1285) = 4.78, *p* = 0.03.

### Factors associated with mental health measures

The descriptive data for the three mental health scales are presented, along with Mann–Whitney *U* test comparisons, as detailed in Table 2.

**TABLE 2:** Physiological distress (K10), depression (PHQ‐2) and anxiety (GAD‐2) scores per population group.

Measure	Median (IQR)	Mean (SD)	% with at risk scores	*N*	*p* Value
*K10 overall*	21 (16–28)	22.2 (8.1)	37%	1291	
Female	22 (17–29)	22.9 (7.9)	39%	861	
Male	19 (14–26)	20.8 (8.3)	31%	417	<0.001
Non‐IPs	22 (16–29)	22.8 (8.2)	38%	1046	
IPs	20 (15–25)	19 (15)	28%	236	<0.001
Additional roles	19 (14–26)	20.9 (7.7)	30%	554	
No additional roles	22 (17–29)	23.2 (8.2)	42%	737	<0.001
Full time (≥35 h/week)	22 (16–29)	22.7 (8.2)	40%	809	
Part time (<35 h/week)	20 (16–26.3)	21.4 (7.8)	31%	482	0.006
Less experience (≤5 years)	24 (18–31)	24.8 (8.3)	49%	320	
More experience (>5 years)	20 (15–27)	21.4 (7.9)	32%	968	<0.001
*PHQ‐2 overall*	1 (0–2)	1.7 (1.6)	24%	1296	
Female	2 (0–3)	1.7 (1.6)	25%	865	
Male	1 (0–3)	1.5 (1.6)	22%	428	0.02
Non‐IPs	2 (0–3)	1.8 (1.7)	26%	1049	
IPs	1 (0–2)	1.3 (1.5)	16%	238	<0.001
Additional roles	1 (0–2)	1.5 (1.6)	21%	557	
No additional roles	2 (0–3)	1.8 (1.7)	26%	739	0.006
Full time (≥35 h/week)	1 (0–3)	1.8 (1.7)	27%	813	
Part time (<35 h/week)	1 (0–2)	1.5 (1.5)	20%	483	0.02
Less experience (≤5 years)	2 (1–3)	2.0 (1.7)	30%	319	
More experience (>5 years)	1 (0–2)	1.6 (1.6)	22%	974	<0.001
*GAD‐2 overall*	2 (0–3)	1.9 (1.8)	28%	1296	
Female	2 (0–3)	2 (1.8)	31%	865	
Male	1 (0–2)	1.6 (1.7)	24%	418	<0.001
Non‐IPs	2 (0–3)	2 (1.9)	30%	1049	
IPs	1 (0–2)	1.5 (1.5)	19%	238	0.004
Additional roles	1 (0–3)	1.7 (1.8)	25%	559	
No additional roles	2 (0–3)	2 (1.9)	31%	737	0.003
Full time (≥35 h/week)	2 (0–3)	2 (1.9)	31%	813	
Part time < (35 h/week)	2 (0–2)	1.7 (1.7)	24%	483	0.08
Less experience (≤5 years)	2 (0–4)	2.3 (1.9)	37%	318	
More experience (>5 years)	1 (0–3)	1.7 (1.8)	23%	975	<0.001

*Note*: At risk scores were classed as ≥25 for K10 (classified as moderate or severe),^14^ and ≥3 for PHQ‐2 or GAD‐2.^29^

Abbreviation: IP, independent prescriber.

Individuals who adhered to recommended healthier lifestyle behaviours generally demonstrated better scores on mental health measures compared to those who did not. Mann–Whitney *U* tests revealed that non‐smokers exhibited significantly lower scores on all three mental health measures compared to smokers, with statistical significance across all measures (*p* < 0.001). In contrast, no significant differences were observed for alcohol consumption with respect to any of the mental health measures (*p* > 0.05). Regarding dietary and exercise habits, adherence to fruit/vegetable consumption guidelines was associated with lower scores on the K10 (*p* = 0.001) and PHQ‐2 (*p* = 0.008). Similarly, engagement in physical exercise was linked to lower scores on the PHQ‐2 (*p* = 0.008).

Kruskal–Wallis tests showed no significant differences across the workplace groups (Multinational chain, Independent, Hospital or Locum optometrist) for K10 (*X* ^2^ (3) = 5.82, *p* = 0.12), PHQ‐2 (*X* ^2^ (3) = 4.79, *p* = 0.19), or GAD‐2 scores (*X* ^2^ (3) = 2.12, *p* = 0.55) and no significant differences across areas of work (England, Wales, Scotland and Northern Ireland) for K10 (*X* ^2^ (3) = 1.62, *p* = 0.65), PHQ‐2 (*X* ^2^ (3) = 3.49, *p* = 0.32) or GAD‐2 scores (*X* ^2^ (3) = 2.77, *p* = 0.43).

### Prediction models

Logistic regression analysis (Table 3) identified age, physical health self‐rating and additional roles as the strongest predictors of moderate‐to‐severe psychological distress (K10 ≥ 25) among participating optometrists (*N* = 1270). Each additional year of age reduced the odds by 3% (OR = 0.97, *p* < 0.001). Higher physical health ratings decreased the odds by 26% per unit increment (OR = 0.74, *p* < 0.001). Optometrists without additional roles had 38% higher odds of distress than those with additional roles (OR = 1.38, *p* = 0.01).

**TABLE 3:** Predictors among optometrists of moderate–severe distress (K10), positive depression outcome (PHQ‐2) and positive anxiety score (GAD‐2).

Measure	Predictor	Coefficient	*p* Value	Odds ratio	95% CI	
					Lower	Upper
K10	Age	−0.036	<0.001	0.97	0.95	0.98
*N* = 1270	Physical health rating	−0.299	<0.001	0.74	0.69	0.80
	Additional roles	0.325	0.01	1.38	1.08	1.78
PHQ‐2	Age	−0.023	<0.001	0.98	0.97	0.99
*N* = 1268	Physical health rating	−0.259	<0.001	0.77	0.71	0.83
	IP status	0.507	0.01	1.66	1.13	2.44
GAD‐2	Age	−0.029	<0.001	0.97	0.96	0.98
*N* = 1268	Physical health rating	−0.216	<0.001	0.81	0.75	0.87
	IP status	0.444	0.01	1.56	1.09	2.23

*Note*: The degrees of freedom were 1 for all variables, and *R* ^2^ for K10 = 0.13, PHQ‐2 = 0.08 and GAD‐2 = 0.08.

*Abbreviation*: IP, independent prescriber.

For depression and anxiety (*N* = 1268), age, physical health self‐rating and IP status were the strongest predictors. Each additional year of age reduced the odds by approximately 2% for depression (OR = 0.98, *p* < 0.001) and 3% for anxiety (OR = 0.97, *p* < 0.001). Each unit increase in physical health rating reduced the odds of depression by 23% (OR = 0.77, *p* < 0.001) and anxiety by 19% (OR = 0.81, *p* < 0.001). Non‐IP optometrists had 66% higher odds of depression (OR = 1.66, *p* = 0.01) and 56% higher odds of anxiety (OR = 1.56, *p* = 0.01).

## DISCUSSION

This research represents the first formal exploration of mental health and well‐being within the UK optometry profession. Approximately 17% of optometrists reported receiving treatment for a mental health condition within the past year. In total, 37% were classified as having moderate‐to‐severe psychological distress scores (K10 ≥ 25), while 24% and 28% screened positive for depression (PHQ‐2 ≥ 3) and anxiety (GAD‐2 ≥ 3), respectively. Older age and higher self‐rated physical health were the strongest predictors of more favourable outcomes across all three mental health measures in the regression models. Holding an additional role was the third strongest predictor of lower psychological distress, while IP status predicted both lower anxiety and depression scores.

Compared with the general population, psychological distress and anxiety were significantly elevated among UK optometrists, while depression levels were broadly similar.^29^ The proportion of participants with moderate‐to‐severe psychological distress scores was consistent with a large national study of adults in Wales (*n* = 12,415) during the peak of the COVID‐19 pandemic, where 37% met the threshold.^30^ This period represented a national peak in prevalence, with a 3‐4 fold increase in severe K10 scores compared to pre‐pandemic 2019 levels.^30^ Longitudinal studies in the general population suggest that the prevalence of common mental health conditions is now returning towards pre‐pandemic levels.^52^, ^53^

International comparisons provide further context. UK optometrists had significantly higher moderate‐to‐severe psychological distress scores (K10 ≥ 25) than their Australian counterparts (37% vs 31%) and similar to Australian doctors (39%).^54^ However, these comparisons should be interpreted with caution, as the Australian data were collected prior to the COVID‐19 pandemic. Additional factors may include the psychological effect of the Honey Rose manslaughter case,^19^ as well as systemic differences in professional practice, such as less uptake of independent therapeutic prescribing in the UK.^55^ The prevalence of depression and anxiety among Australian optometrists cannot be directly compared to our findings due to the study's use of the Depression Anxiety Stress Scale (DASS‐21), which differs from the PHQ‐2 and GAD‐2 used in this study. The PHQ‐2 and GAD‐2 were employed in a longitudinal study of ophthalmic personnel in the USA and Canada, conducted during the COVID‐19 pandemic.^15^ That study reported lower prevalence of depression (PHQ‐2 ≥ 3) compared to the UK (18% vs 24%, respectively) and higher prevalence for anxiety (GAD‐2 ≥ 3: 35% vs 28%). However, these comparisons should also be interpreted with caution due to differences in sample composition; the study in the USA and Canada included 42% optometrists, 37% students, 12% ophthalmologists and 9% support staff.^15^ The broader context of the pandemic should also be considered. Lastly, a recent study of American Academy of Optometry Fellows conducted post‐COVID‐19 reported lower prevalence of depression (PHQ‐2 ≥ 3: 4% vs 24%) and anxiety (GAD‐2 ≥ 3: 9% vs 28%) than observed in this study. However, nearly half of the participants (46% of 321) worked in academic settings and 74% were aged 40 years or older.^21^ In contrast, our study only included practising, patient‐facing optometrists, of whom only 48% were aged 40 years or older. Additionally, there are substantial differences in the scope of optometric practice in the UK compared with the USA and Canada.^56^

Psychological distress has also been reported among other healthcare providers internationally. A study of 272 Australian medical practitioners during the COVID‐19 pandemic found that 73% experienced psychological distress, as defined by a K10 score of ≥16. When the same K10 cut‐off is applied to our data, 77% of UK optometrists reported psychological distress. These figures are higher than the distress rate observed in a longitudinal study of Canadian healthcare providers^57^ (physicians, nurse practitioners and midwives), in which 49% of the 415 respondents reported psychological distress (K10 ≥ 16) in 2023. This represented a 20% decrease from the 69% reported during the peak of the pandemic in 2021.^57^ There is an absence of studies that directly compared optometrists to other healthcare professionals both in the UK and worldwide.

Several factors were linked to mental well‐being. In the general UK population, females have been found to be more likely than males to self‐report mental health issues, with young adults (aged 16–24 years) being at the greatest risk compared to all other age groups.^58^ In this study, younger age was identified as a stronger predictor of poorer mental health than fewer years since qualification or female gender, across all three measures (K10, PHQ‐2 and GAD‐2). The association between poorer self‐rated physical health scores and worse mental health outcomes aligns with existing literature, which indicates that adults in good physical health report significantly higher life satisfaction and lower mental health issues compared to those in poor physical health.^22^ Previous research involving healthcare professionals reported similar self‐rating health scores^28^ and studies among nurses have shown that those with lower self‐rated physical or mental health scores are more prone to making medical errors.^48^

The associations between optometrists with additional roles or qualifications (such as IP) and better mental health‐related scores may support theories linking low control at work with mental health issues,^59^ or may reflect that optometrists with mental health issues are less likely to take on additional roles or training. Longitudinal and qualitative studies are needed to avoid assumptions and to better understand these findings. Tobacco use was the only unhealthy lifestyle behaviour significantly associated with all three mental health measures, although interpretation is limited due to the small proportion of smokers (8%). The relationship between unhealthy behaviours (e.g. smoking, excessive alcohol consumption and unhealthy eating) and psychological issues is complex and likely bidirectional.^60^ While optometrists who adhered to healthy lifestyle behaviours generally reported more favourable mental health scores, these behaviours were not retained as independent predictors in the final logistic regression model. It is unclear whether unhealthy behaviours act as coping mechanisms or contribute to psychological distress.^61^, ^62^

### Implications for clinical practice and future research

This study offers important insights into the mental health status of optometrists practising in the UK. In the past year, 21% of UK optometrists had sought professional mental health support, a figure comparable to those reported for UK National Health Service (NHS) healthcare workers (20%)^63^ and Australian optometrists (26%).^14^ However, nearly one in three optometrists (31%) with moderate‐to‐severe psychological distress scores had not accessed professional support, underscoring the need to better identify and assist those at risk, particularly where barriers to help‐seeking exist.

Those at greater risk, such as younger optometrists, female optometrists and individuals reporting poorer physical health, may benefit from targeted support. Higher qualifications and greater professional responsibilities were associated with reduced risk, independent of age. These findings suggest that expanding access to professional development, role diversification and structured career progression could offer protective benefits, though this hypothesis warrants further investigation. Such engagement may be especially relevant for early career professionals, among whom approximately half (49%) reported moderate‐to‐severe psychological distress.

Based on these findings, the authors recommend the following priorities for clinical practice. First, greater awareness of mental health risk factors should be promoted across the profession. Second, clearer signposting to support services and open dialogue about well‐being should be normalised within practice teams. Third, all stakeholders should integrate mental well‐being initiatives to help foster a positive and healthy workforce culture, although the effectiveness of such initiatives requires further evaluation.

Further research is needed to explore the association between the identified factors and mental health outcomes. Longitudinal studies could clarify whether additional responsibilities improve well‐being, or whether individuals with better mental health are more likely to pursue them. Qualitative research would also provide valuable insight into barriers to help‐seeking and the lived experiences of optometrists at different career stages, helping to inform the development of targeted, evidence‐based interventions.

### Limitations

This study used two brief mental health screening measures (PHQ‐2 and GAD‐2), with only two questions each. These were chosen for their brevity, to encourage participants to fully complete all aspects of the questionnaires. However, this may also be viewed as a limitation, as these questionnaires are designed as a screening tool only, with the intention of follow‐up exploration for those with a score above the 3‐mark threshold. These screening tools do not fully explore mental health status and do not provide a definitive diagnosis. There is also likely to be a sampling bias, as individuals experiencing mental health challenges may be more likely to participate in questionnaires of this nature. For example, this may account for the 17% of optometrists who had received mental health treatment within the last year. However, all questionnaire‐based studies in this area are likely to encounter similar sample bias due to reliance on voluntary sampling.^14^, ^15^, ^21^, ^58^ To mitigate limitations, anonymised response forms were used, and a broad awareness campaign was implemented to encourage wide representation across the profession.

Assumptions should be avoided when comparing different groups or studies from differing timeframes, particularly given the absence of longitudinal data in the present study. While observed differences seem plausible, certain groups may have an inherent predisposition towards better mental health. Furthermore, the absence of data on participants' ethnicity represents a potential confounding factor, as mental health differences across ethnic groups have been observed in the UK general population.^58^ Longitudinal and mixed‐method studies could provide deeper insights into these differences and address the limitations identified.

## CONCLUSION

This study identified elevated levels of psychological distress and anxiety, as well as comparable levels of depression, among UK optometrists relative to the general adult population.^29^ Risk factors for poorer mental well‐being included younger age, female, early career and lower self‐rated physical health, while protective factors were associated with higher qualifications, additional professional roles and healthier lifestyle choices. These findings highlight the need for targeted mental well‐being initiatives within the profession, particularly those focused on supporting individuals most at risk. Further research is warranted to develop and evaluate effective interventions tailored to the needs of the UK optometry profession.

## AUTHOR CONTRIBUTIONS

**Neil Retallic:** Conceptualization (lead); data curation (lead); formal analysis (equal); investigation (lead); methodology (equal); project administration (lead); validation (equal); writing – original draft (lead); writing – review and editing (equal). **Lindsay Rountree:** Conceptualization (supporting); funding acquisition (equal); investigation (supporting); methodology (equal); supervision (supporting); validation (equal); writing – review and editing (equal). **Sharon Bentley:** Conceptualization (supporting); methodology (equal); validation (equal); writing – review and editing (equal). **Fiona Fylan:** Conceptualization (supporting); methodology (equal); validation (equal); writing – review and editing (equal). **David B. Elliott:** Conceptualization (supporting); formal analysis (equal); funding acquisition (equal); investigation (supporting); methodology (equal); supervision (lead); validation (equal); writing – original draft (supporting); writing – review and editing (equal).

## FUNDING INFORMATION

This study was funded by a College of Optometrists research grant.

## CONFLICT OF INTEREST STATEMENT

The authors report no conflicts of interest.
